# Interdigital Toe Web Space Erythrasma in a Young Child: A Case Report

**DOI:** 10.7759/cureus.48435

**Published:** 2023-11-07

**Authors:** Mara Trifoi, Shayan Waseh, Sylvia Hsu

**Affiliations:** 1 Dermatology, Pennsylvania State University College of Medicine, Hershey, USA; 2 Dermatology, Temple University Hospital, Philadelphia, USA

**Keywords:** wood's lamp, clinical dermatology, cutaneous infections, pediatrics, erythrasma

## Abstract

Erythrasma is a common superficial skin infection in adults. However, there is a paucity of reported cases in the pediatric population. Here we report a case of interdigital pedal erythrasma presenting in a four-year-old child as itchy, scaly maceration.

## Introduction

Erythrasma is a common superficial cutaneous bacterial infection caused by the gram-positive bacillus Corynebacterium minutissimum. The condition most frequently presents in adults as hyperpigmented or erythematous well-defined patches localized to moist intertriginous areas, including the axillae, inframammary skin, genitocrural creases, or interdigital toe web spaces. Interdigital erythrasma is the most common bacterial infection of the foot, and the most frequent form of erythrasma [1]. The cutaneous manifestations may be accompanied by itching, scaling or may be asymptomatic [2]. Excessive moisture and occlusion are thought to lead to overgrowth of the bacterium and invasion into the stratum corneum [3]. Patients with diabetes, advanced age, inverse psoriasis, obesity, hyperhidrosis, hidradenitis suppurative, and those who live in warmer climates are more prone to infection [4,5]. Despite its common incidence, erythrasma is relatively uncommon in the pediatric population, making it a diagnostic challenge. We highlight a presentation of erythrasma as itchy scaling and maceration of the interdigital toe web spaces in a young child with obesity.

## Case presentation

The patient was a four-year-old male child with obesity who presented to the dermatology clinic accompanied by his father with complaints of persistent pruritus between his toes for the past two months. There was no prior history of such symptoms, and the patient had no recent travel history or changes to his medical status. Additionally, no one in the household complained of similar symptoms. 

**Figure 1:**
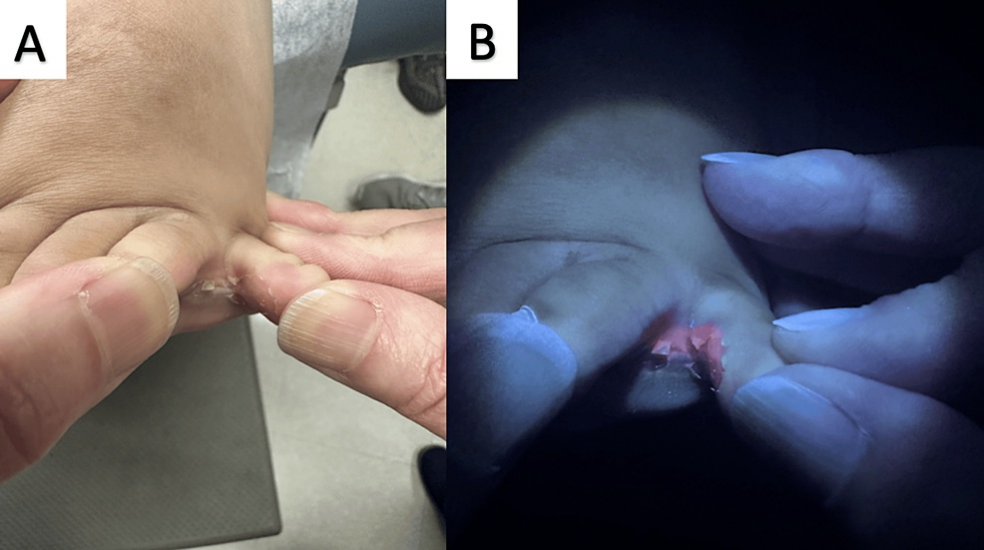
Interdigital erythrasma of the fourth toe web space under fluorescence examination A. Scaling and maceration of the fourth interdigital toe web space. B. Positive coral red fluorescence on Wood’s lamp examination.

On clinical examination, the patient had scaling and maceration in all of his interdigital toe web spaces bilaterally (Figure 1). Examination with a Wood’s lamp revealed bright coral-red fluorescence corresponding to these same areas of maceration and scale. In light of the pathognomonic, positive Wood's lamp test, a KOH preparation test or bacterial culture were not deemed necessary. The child was subsequently treated with clindamycin 1% lotion daily for two weeks with complete resolution of his symptoms.

## Discussion

Erythrasma primarily presents in adult patients, affecting moist intertriginous regions, such as the axillae, inframammary skin, genitocrural creases, or interdigital toe web spaces. Its presentation in pediatric patients is much less frequent and therefore may be more easily missed or misdiagnosed [6,7]. Misdiagnosis of erythrasma in pediatric patients may lead to ineffective treatment and prolonged discomfort. Given its relatively infrequent presentation in pediatric populations, erythrasma may be confused as a localized expression of atopic dermatitis, such as juvenile plantar dermatosis, or as a dermatophyte infection, such as tinea pedis. 

Therefore, an essential component of the evaluation of interdigital toe web space scale and maceration in pediatric populations is the Wood’s lamp examination, which allows for rapid and sensitive identification of Corynebacterium minutissimum through the coral-red fluorescence of coproporphyrin III. Culturing of the bacterium is difficult, requiring special media requirements, and is not routinely performed. Treatment of erythrasma most often consists of topical therapy with erythromycin or clindamycin, but oral macrolides, such as clarithromycin or erythromycin have also been reported to be effective [5].

## Conclusions

Pediatric cases of erythrasma are rare in comparison to adult populations. Given its infrequent presentation in children, erythrasma may be more easily missed or misdiagnosed. Therefore, the use of Wood’s lamp examination in children with maceration or scaling of the interdigital toe web spaces is essential.

## References

[1] Garcia-Souto F (2020). Visual dermatology: erythrasma fluorescence under Wood’s lamp. J Cutan Med Surg.

[2] Groves JB, Nassereddin A, Freeman AM (2022). Erythrasma. StatPearls.

[3] Blaise G, Nikkels AF, Hermanns-Lê T, Nikkels-Tassoudji N, Piérard GE (2008). Corynebacterium-associated skin infections. Int J Dermatol.

[4] Polat M, I˙lhan MN (2015). Dermatological complaints of the elderly attending a dermatology outpatient clinic in Turkey: a prospective study over a one-year period. Acta Dermatovenerol Croat.

[5] Forouzan P, Cohen PR (2020). Erythrasma revisited: diagnosis, differential diagnoses, and comprehensive review of treatment. Cureus.

[6] Halpern AV, Heymann WR (2008). Bacterial disease. Dermatology, 2nd ed.

[7] Karakatsanis G, Vakirlis E, Kastoridou C, Devliotou-Panagiotidou D (2004). Coexistence of pityriasis versicolor and erythrasma. Mycoses.

